# Isolation and characterization of thermophilic cellulose and hemicellulose degrading bacterium, *Thermoanaerobacterium* sp. R63 from tropical dry deciduous forest soil

**DOI:** 10.1371/journal.pone.0236518

**Published:** 2020-07-23

**Authors:** Phurt Harnvoravongchai, Ratiyakorn Singwisut, Puey Ounjai, Amornrat Aroonnual, Pahol Kosiyachinda, Tavan Janvilisri, Surang Chankhamhaengdecha

**Affiliations:** 1 Department of Biology, Faculty of Science, Mahidol University, Bangkok, Thailand; 2 Department of Tropical Nutrition and Food Science, Faculty of Tropical Medicine, Mahidol University, Bangkok, Thailand; 3 Department of Biochemistry, Faculty of Science, Mahidol University, Bangkok, Thailand; Purdue University, UNITED STATES

## Abstract

Thermophilic microorganisms and their enzymes have been utilized in various industrial applications. In this work, we isolated and characterized thermophilic anaerobic bacteria with the cellulose and hemicellulose degrading activities from a tropical dry deciduous forest in northern Thailand. Out of 502 isolated thermophilic anaerobic soil bacteria, 6 isolates, identified as *Thermoanaerobacterium* sp., displayed an ability to utilize a wide range of oligosaccharides and lignocellulosic substrates. The isolates exhibited significant cellulase and xylanase activities at high temperature (65°C). Among all isolates, *Thermoanaerobacterium* sp. strain R63 exhibited remarkable hydrolytic properties with the highest cellulase and xylanase activities at 1.15 U/mg and 6.17 U/mg, respectively. Extracellular extract of *Thermoanaerobacterium* sp. strain R63 was thermostable with an optimal temperature at 65°C and could exhibit enzymatic activities on pH range 5.0–9.0. Our findings suggest promising applications of these thermoanaerobic bacteria and their potent enzymes for industrial purposes.

## Introduction

Lignocellulosic biomass is considered as one of the most abundant and inexpensive renewable resources on earth. Estimation of 200 billion tons of lignocellulosic biomass are produced annually from both agricultural and industrial sectors [1]. A great number of research has been conducted to develop efficient means to convert biomass to high value products [2–5]. However, the recalcitrant nature of lignocellulosic biomass has presented a major obstacle for conversion and utilization of cellulosic biomass. Although different physical and chemical methods for breaking down cellulosic substances have been established to effectively hydrolyze complex cellulosic polymers, physicochemical treatments often require harsh and extreme conditions [6]. Biological treatments are, thus, considered a promising approach for utilizing cellulosic waste because most of the isolated microbial enzymes can often catalyze hydrolytic reactions under ambient environment.

In nature, two key enzymes that play vital roles in plant biomass degradation are cellulase and hemicellulase [7–9]. Cellulase is a group of enzymes that catalyze the hydrolysis of β-1,4 glycosidic bond of cellulose. Cellulase enzymes can be classified into three groups: endoglucanase, exoglucanase, and β-glucosidase based on their specific hydrolytic sites [10]. Hemicellulases catalyze depolymerization of hemicellulose into smaller units. Such enzymes are categorized based on their substrates, for example, xylanase for xylan degradation [11]. These cellulolytic enzymes have been widely and extensively exploited in different industries including food, textile, paper, animal feeds, and biofuel [12–14].

Microorganisms have been widely investigated for their potential lignocellulosic biomass degrading properties [15–17]. Although several enzymes have been identified and studied, implementation of these enzymes is often limited to mesophilic conditions, moderate functional temperature range, and mild pH conditions. An enzyme with greater durability and stability would be preferred in industrial settings for lignocellulosic biomass hydrolysis [18,19]. Therefore, tremendous research effort has been continuously put to seek for novel thermophilic biomass degrading enzymes from different environment.

Tropical forests are known to have the highest biodiversity in the world [20]. Tropical dry deciduous forest is of particular interest as it possesses unique ecological and biological diversity. The condition of such ecosystem is often known to promote not only the enrichment of endemism but also species richness. In this work, novel thermophilic enzymes were screened from rare environmental conditions of the tropical dry deciduous forest in Thailand for their cellulose and hemicellulose degrading abilities. Further characterization of these enzymes under various temperature and pH ranges suggested thermostability and wide working ranges of the enzymes and, thus, highlighted their potential industrial applications.

## Materials and methods

### Sample collection, bacterial isolation, and screening of cellulose-degrading isolates

Soil samples were collected at approximately 30 cm depth from three different sites of a tropical dry deciduous forest of Nan province, Thailand. Sample collecting sites in this study are located in the boundary area of national park, no specific permissions were required for these locations. The samples were immediately subjected to anaerobic jars to maintain anaerobic condition, transferred to laboratory within 24 h, and stored at 4°C. To avoid loss in microbial diversity, the samples used for bacterial isolation were manipulated within 72 h.

One gram of soil sample was suspended in 9 ml of 0.85% saline solution and incubated at 60°C for 1 h. After the suspension was cooled at room temperature, a serial dilution was performed. The sample was spread on modified T6 agar supplemented with 1.5% carboxymethylcellulose (CMC). T6 medium contained (g/L): tryptone, 6.0; yeast extracts, 2.0; NH_4_CH_3_COO_2_, 3.0; KH_2_PO_4_, 0.5; MgSO_4_.7H_2_O, 0.3; FeSO_4_.7H_2_O, 0.01. The plates were, then, incubated at 60°C under anaerobic condition for 2 days. To acquire homogenous population of bacteria, a single colony was resuspended into 10 ml of pre-reduced 0.85% saline solution and incubated at 60°C for 1 h to eliminate mesophilic bacteria contamination. Subsequently, cooled suspension was serially diluted and plated was on modified T6 agar supplemented with 1.5% CMC. Purification process was repeated for 5 times to obtain axenic culture. For screening of potential cellulose-degrading bacteria, Gram's iodine method was carried out as described previously [21]. The hydrolyzing capacity (HC) value was calculated from the ratio between the halo zone and the bacterial colony diameter. The HC value was used to determine hydrolytic activity of each isolate.

### Identification of bacterial isolates and taxonomic classification

Total DNA of selected bacterial isolates were extracted using bacterial DNA kit (OMEGA, Bio-tek). The universal primers of UFUL (5'–GCCTAACACATGCAAGTCGA–3') and 800R (5'–TACCAGGGTATCTAATCC–3') were used to amplify partial 16S rRNA gene. PCR products were subjected to DNA sequencing. All sequences were deposited in the GenBank database under accession numbers MT573539, MT578048, MT578049, MT573879, MT573881, and MT573882. The nucleotide sequences were queried in EZBioCloud [22] server using Basic Local Alignment Search Tool (BLAST). Phylogenetic trees for subsequent analysis were obtained using MEGA7 with neighbor-joining method. All sequences were aligned using CLUSTALW, and the confidence level of tree topology was estimated with 1,000 replicates bootstrap re-sampling analysis.

### Substrate utilization and lignocellulosic biomass degradation

To determine the ability of the bacterial isolates in utilization of different oligosaccharides and complex substrates, bacterial isolates were cultured in T6 medium containing 0.72% phenol red with the initial pH adjusted to 7.0. The media were supplemented with 1% of selected carbohydrate substrate. The cultures were incubated at 60°C for 2 days. Acid production from the fermentation process was monitored by observation on the color changing from orange red to yellow of the phenol red indicator.

For biomass degradation assay, bacterial isolates were enriched in T6 medium supplemented with 1% glucose for 24 h. The bacterial cells were harvested by centrifugation at 5,000 x g for 20 min; then, washed twice with T6 medium to avoid carried-over glucose. Harvested bacterial pellets were resuspended in 50 ml of T6 medium containing 5% of cassava pulp, Napier grass, or bark. After that, fermentation was performed at 60°C under anaerobic condition for 7 days. The remaining substrates were simultaneously filtered and dried at 65°C for 3 days before determining quantity of the remaining mass. To investigate a non-bacterial degradation, a control experiment was conducted by incubating each of the substrate in 50 ml of T6 medium at 65°C for 7 days. The total weight loss was calculated by subtracting the measured dry weight from that of initial substrate and non-bacterial degradation.

### Enzymatic activity assay

Crude enzymes were prepared from bacterial culture grown for 24 h in T6 medium supplemented with 2% CMC, xylan (from corn core with the minimum of 75% xylose after hydrolysis, Tokyo Chemical Industry), starch, crystalline cellulose (Avicel), or 0.5% filter paper as the sole carbon source. The crude enzymes were pre-incubated at 60°C for 5 min. Enzymatic reactions were initiated by adding 1 ml of the crude enzyme into equivalent volume of 2% (w/v) of corresponding substrates dissolved in 50 mM sodium citrate buffer (pH 5.0). The reactions were carried out at 60°C for 60 min and terminated by adding 3 ml of DNS reagent. For the control, the reaction mixture was incubated in slurry ice for 60 min instead. The sample was, then, heated in boiling water for 5 min for color development. The specimen was cooled down on ice for 5 min, and the amount of reducing sugars released from the reaction was determined by measuring the absorbance at 540 nm. Total protein concentration was estimated using Bradford protein assay [23]. One unit (U) of the enzyme activity was defined as the amount of enzyme used to produce one μmol of reducing sugars per minute under optimal assay condition.

### Growth determination

To investigate the optimal growth condition of *Thermoanaerobacterium* sp. R63, 5% of an overnight culture of cell suspension was inoculated into pre-reduced T6 medium supplemented with 1% glucose. To determine the range of temperature for bacterial growth, bacterial isolates were grown at 37, 45, 50, 55, 60, and 65°C with initial pH of 7.0 under anaerobic condition. Bacterial growth was followed by monitoring the optical density at 600 nm at 3 h interval for 54 h. For pH effect, *Thermoanaerobacterium* sp. R63 was grown under different ranges of pH. For acidic media, T6 medium with 1% glucose was prepared in 50 mM sodium citrate buffer, and the pH was adjusted to the initial pH of 4.0, 5.0, and 6.0. T6 medium with 1% glucose with initial pH of 7.0 and 8.0 were prepared in phosphate buffer, while glycine/NaOH was used to obtain the medium with initial pH of 9.0. Bacterial growth was determined by monitoring optical density at 600 nm every 3 h up to 54 h.

### Effects of temperature and pH on enzyme stability and activity

To determine the effect of temperature and pH on stability and activity of crude enzyme, the enzymatic reactions were carried out by mixing the equal volume of crude enzyme and 100 mM sodium citrate buffer, pH 5.0, supplemented with either 2% CMC or 2% xylan. The reactions were allowed to occur at various temperatures from 37°C to 70°C for 30 min. Cellulase and xylanase activities were independently measured using DNS method. Furthermore, the effects of pH were also assayed by incubating the crude enzymes with substrate in a series of buffers representing different pH ranges as follows: sodium citrate buffer (pH 4.0–5.0), phosphate buffer (pH 7.0–8.0), and glycine/NaOH (pH 9.0). The reactions were carried out under the optimal temperature for 30 min. Hydrolytic activities of the crude enzymes were calculated.

To address thermostability of the crude enzyme, 1 ml of 100 mM sodium citrate buffer, pH 5.0 was incubated at various temperatures from 37°C to 85°C for 1 h before setting up the reaction. The reactions were initiated by addition of 1 ml of crude extract followed by incubating the solution for 1 h at a corresponding temperature. Residual hydrolytic activity was examined using DNS method. For pH stability, the crude enzyme was incubated in different buffers, prepared at different pH ranging from 3.0–10.0 for 1 h. The remaining hydrolytic activity of the enzyme was further evaluated using DNS method under the optimal temperature.

### High-performance liquid chromatography analysis

To analyze hydrolytic products, crude enzymes were harvested from *Thermoanaerobacterium* sp. R63 cultured at 42 and 54 h for xylanase and cellulase, respectively. Mixtures of enzyme and substrate at the ratio of 1:2 were incubated for 3 h under the optimal conditions. The hydrolytic products were, then, concentrated with evaporation and harvested with centrifugation. High-performance liquid chromatography (HPLC) was carried out using Water Alliance 2690 HPLC Separation Module with a ZOBAX Carbohydrate analysis column (4.6x250 mm) with an injection volume of 10 μl using 70% acetonitrile as mobile phase at flow rate of 1 ml/min. The linearity of calibration curve was quantified by injecting the mixture of glucose, xylose, and cellobiose solutions at 0.2, 0.4, 0.6, 0.8, and 1.0 mg/ml. The calibration curve for each sugar was calculated by plotting concentration of the compound against the area of the respective peaks.

### DNA extraction and metagenomic analysis

Total genomic DNA was extracted from five grams of the soil sample collected from the third sampling site with DNeasy PowerSoil Pro Kit (QIAGEN) according to the manufacturer’s instructions. All the qualified DNA was used to construct a library of fragments amplification of the V3 and V4 regions of the 16S rRNA gene. Then, the products were used for Illumina library preparation, and the processed libraries were performed on the Illumina MiSeq platform (BGI Genomics Co., Ltd., Hong Kong). Functional analysis was predicted using PICRUSt to evaluate host-associated and environmental community based on KEGG Orthology (KOs) [24].

### Statistical analysis

Statistical analysis was assessed by ANOVA with post-hoc Tukey’s multiple comparison test using GraphPad Prism 8.4.2.

## Results and discussion

### Isolation of thermophilic cellulolytic microbes

Accumulation of leaf littering is a unique characteristic of tropical dry deciduous forest [25]. To recycle carbon and nutrients, decomposition of litter is developed, and this is usually completed by microorganisms [26]. Consequently, tropical dry deciduous forest could provide a library of thermophilic microorganisms that could be a great reservoir of thermostable enzymes of industrial significance. According to the data of Royal Forest Department of Thailand in 2006, deciduous forest was estimated to be the second most abundant type of forest in Thailand with the area approximately of 11,000,000 ha, and most were located in the northern region of the country. Nan province, located in northern Thailand, is covered by the largest area of deciduous forest at 667,193 ha [27]. Here, a total number of 502 thermophilic anaerobic bacteria were obtained from soil samples collected from three different sites of tropical dry deciduous forest in Nan province. Soil samples from the first and the second sites were collected from base of the fallen dead tree trunk. Both soil samples have dark brown color with a loamy texture. The third collection site was from a pile of decaying leaves, where the soil characteristic is clay loam with light brown color.

The screening for cellulase activity resulted in 111 isolates with ability to exhibit halo zone on CMC plates. The HC value of CMC was calculated and used to represent cellulase activities. Six bacterial isolates, R13, R21, R28, R53, R63, and R66, were selected for further characterization based on their colony morphological differences on CMC agar, and their high cellulolytic activity (HC value, as summarized in Table 1). Ratio of halo zone diameter to colony diameter or HC value has been used to determine lignocellulosic degradation ability of the bacteria [28–31]. Determination of HC value is, therefore, more practical to screen for lignocellulosic degrading bacteria among a large sample size, when compared to the enzymatic assay. High HC values often indicate high potential lignocellulosic degrading activity because the greater hydrolytic zone over the colony diameter is produced. Regarding to the sampling sites, R13 and R21 were isolated from the first and the second sites, respectively. While the other four isolates; R28, R53, R63, and R66 were retrieved from the third sampling site. It should be noted that the soil from the third sampling site yielded the highest number of potential lignocellulolytic bacterial isolates. The isolates from this particular sample also displayed significantly higher lignocellulosic degrading abilities compared to that of the other two sampling sites; therefore, the soil from the third collecting site was further subjected to metagenomic analysis.

**Table 1:** Hydrolytic activities of bacterial isolates against CMC, xylan, and starch.

Isolate	CMC			Xylan			Starch		
	Colony diameter (cm)	Halo zone (cm)	HC value^1^	Colony diameter (cm)	Halo zone (cm)	HC value^1^	Colony diameter (cm)	Halo zone (cm)	HC value^1^
**R13**	1.62±0.1	15.50±1.0	9.73±0.5	1.52±0.1	16.35±1.0	10.93±0.6	0.31±0.2	ND^2^	-
**R21**	1.58±0.1	14.65±0.4	9.65±0.9	0.38±0.2	ND^2^	-	1.25±0.0	19.18±0.8	15.38±0.8
**R28**	1.25±0.1	15.38±0.3	12.43±0.5	1.70±0.1	19.08±0.8	11.39±0.7	1.25±0.1	13.63±0.5	11.16±1.0
**R53**	1.78±0.1	29.00 ±1.0	16.64±1.4	0.73±0.1	ND^2^	-	1.00±0.0	9.65±0.6	9.65±0.6
**R63**	1.40±0.1	27.73±0.5	20.53±1.8	1.40±0.1	27.72±0.8	20.08±1.2	1.12±0.0	18.75±0.5	16.84±0.6
**R66**	1.18±0.1	27.98±0.6	24.04±1.4	1.38±0.1	27.38±0.4	20.22±1.3	1.07±0.0	17.98±0.6	16.90±0.6

Hydrolytic capacities of six bacterial isolates were calculated from ratio of halo zone over colony diameter. The assay was carried out in duplicate with three independent experiments. ± represents standard error of mean.

^1^HC value: hydrolytic capacity represents ratio of clear zone diameter to colony diameter

^2^ND: not detected

**Fig 1:**
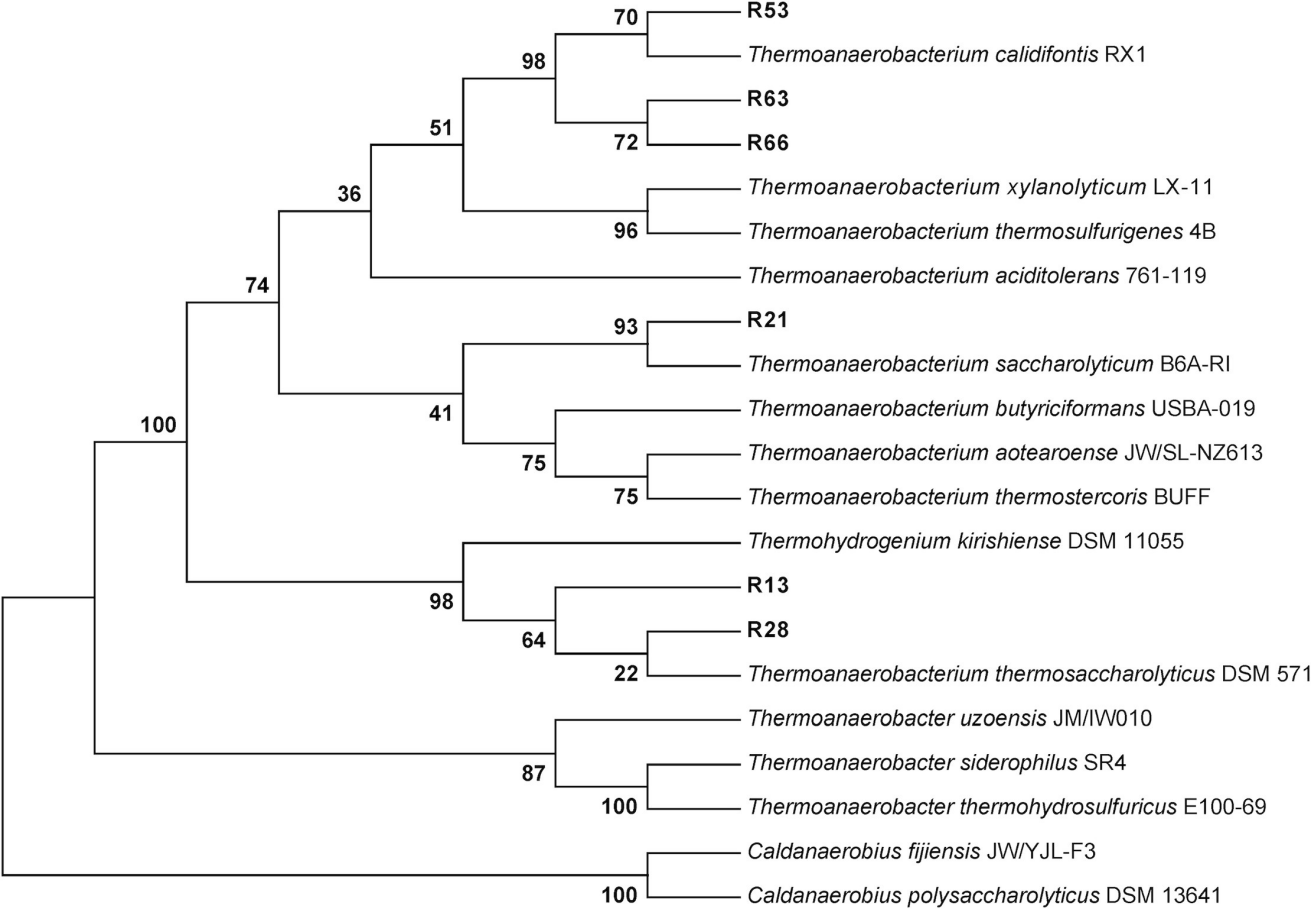
Neighbor-joining tree based on partial sequence of 16S rDNA. The tree showing the phylogenetic relationship of six bacterial isolates (in bold) and closely related thermophilic bacteria. Bootstrap values expressed as percentages from 1,000 replications are located at each node.

Sequence analysis of 16S rDNA revealed that all 6 isolates are closely related to genus *Thermoanaerobacterium*. Phylogenetic analysis suggested that the isolates were clustered into 3 distinct groups as shown in Fig 1. The isolate R53, R63, and R66 were grouped with *T*. *calidifontis* RX1, which was isolated from a hot spring in China [32]. The position of R21 in the phylogenetic tree suggested that it is related to *T*. *saccharolyticum* B6A-RI, which was also originally found at a thermal spring [33]. R13 and R28 shared high sequence similarity with *T*. *thermosaccharolyticus* DSM 571. Most *Thermoanaerobacterium* including other thermophiles such as *Thermoanaerobacter* and *Caldanaerobius* were usually thrived in a hot spring or thermal area. Several previously published works identified lignocellulolytic bacteria including *Bacillus*, *Gordonia*, *Enterobacter*, and *Burkholderia* [31,34,35], as major decomposers in tropical forest. In the current study, however, all isolates belonged to *Thermoanaerobacterium* spp., presumably due to unique selective pressure provided by specific enrichment condition.

### Substrate utilization and hydrolytic activity of *Thermoanaerobacterium*

Bacterial isolates were cultured in T6 medium supplemented with various substrates, and changes of the indicator color specified fermentation in the culture medium. Fermentation of the substrates was summarized in Table 2. Several compounds representing plant biomass were supplied in the medium to examine the ability of bacterial isolates to utilize these plant materials. All *Thermoanaerobacterium* isolates could ferment all C5 and C6 monosaccharides tested in this study. Moreover, all isolates could ferment maltose and cellobiose, but not lactose and sucrose. All isolates were able to ferment carboxymethyl cellulose (CMC), xylan, and starch; however, none of the isolates could utilize lignin as their carbon source. Remarkably, R13, R21, R53, and R63 were able to utilize pectin. Generally, *Thermoanaerobacterium* is able to ferment a wide array of carbohydrate substrates, but the ability to utilize of soluble cellulose; CMC, and insoluble cellulose, Avicel, is not commonly observed in the bacteria of this genus. Most cellulolytic bacteria have been intensively explored in the genus of *Clostridia*. *Clostridium thermocellum*, is the well-studied thermophilic cellulose degrading bacteria in the *Clostridiales* order so far [36].

**Table 2:** Substrate fermentation test of bacterial isolates.

Substrate	Isolate					
	R13	R21	R28	R53	R63	R66
**Glucose**	+	+	+	+	+	+
**Fructose**	+	+	+	+	+	+
**Xylose**	+	+	+	+	+	+
**Arabinose**	+	+	+	+	+	+
**Lactose**	-	-	-	-	-	-
**Sucrose**	-	-	-	-	-	-
**Maltose**	+	+	+	+	+	+
**Cellobiose**	+	+	+	+	+	+
**Maltotriose**	-	-	-	-	-	-
**Pullulan**	-	-	-	-	-	-
**CMC**	+	+	+	+	+	+
**Xylan**	+	+	+	+	+	+
**Starch**	+	+	+	+	+	+
**Pectin**	+	+	-	+	+	-
**Lignin**	-	-	-	-	-	-
**Avicel**	+	+	-	+	+	-
**Bark**	-	-	-	+	-	+
**Napier grass**	+	+	+	+	+	+
**Cassava pulp**	-	+	+	+	+	+

+ indicates development of a yellow color in a culture medium

- indicates no change of a color in a culture medium

**Fig 2:**
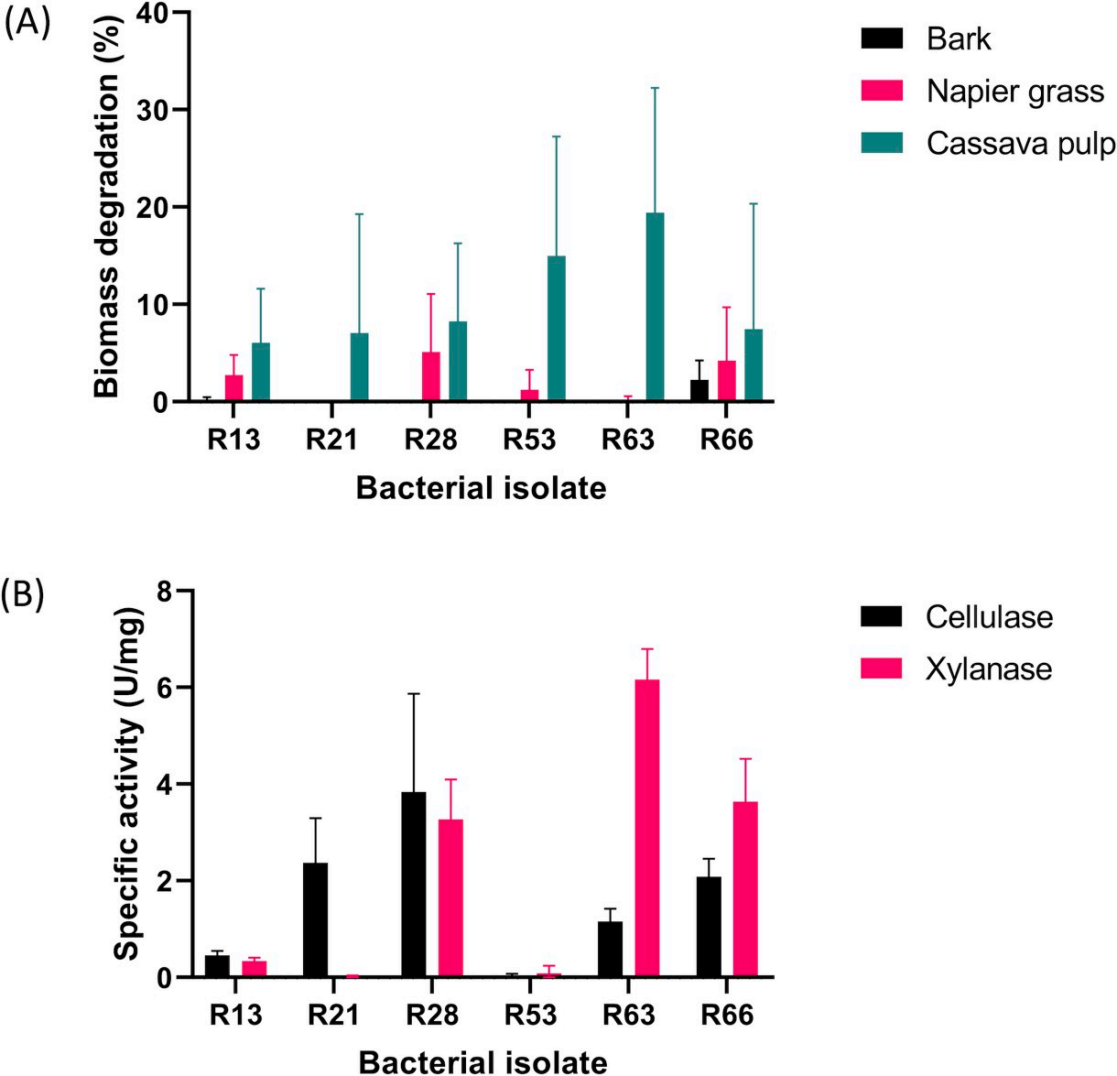
Biomass utilization and enzymatic activity. (A) Profile of biomass degradation of six bacterial isolates against different natural biomass substrates; bark, Napier grass, and cassava pulp after 7 days of incubation at 60°C. (B) Enzyme specificity of the crude enzymes obtained from the six bacterial isolates. Data were evaluated from four independent experiments. Error bars in graphs represent standard deviation. Statistical analysis was determined by two-way ANOVA with post-hoc Tukey’s multiple comparison test (See supplementary in S1 File for detail analysis).

Complex substrates were subjected to the test to further assess the degrading potential of these bacteria (Fig 2A). The results showed that Napier grass and cassava pulp were good carbon sources for most of the *Thermoananerobacterium* isolates; on the other hand, only R53 and R66 could utilize bark as a carbon source. Thus, *Thermoanaerobacterium* isolates from this study have potential to be further developed for biomass fermentation under high temperature condition. Nevertheless, statistical analysis revealed no significant difference of biomass (p < 0.05) degradation between the isolate R53 and R66.

Enzymatic activities of all *Thermoanaerobacterium* isolates were determined against CMC, xylan, Avicel, and starch (Fig 2B and S1 Fig). From the results shown in Fig 2B, extracellular hydrolytic activities of cellulase and xylanase were detected from R28, R63, and R66. The highest xylanase activity was shown in R63, with the reactivity up to 6.17 U/mg. Xylanase activity of *Thermoanaerobacterium* isolates in this study was comparable to those previously reported in *T*. *thermosaccharolyticum* NOI-1 and *T*. *saccharolyticum* JW/SL-YS 485, of which the crude enzyme exhibited 2.55 U/mg and 0.36 U/mg xylanase activity, respectively [37,38]. Furthermore, the crude enzymes of our isolates exhibited up to 100-fold of xylanase activity greater than those report in *T*. *xylanolyticum* B6A-RI (0.065 U/mg) and *T*. *brockii* HTD4 (0.003 U/mg) [36]. R21 possessed only cellulase activity, while a little, if any, cellulase and xylanase activities were detected in R53 and R13 isolates.

Most *Thermoanaerobacterium* and *Thermoanaerobacter* generally possess xylanase activity with non-detectable cellulase activity [36,39–41]. Interestingly, all *Thermoanaerobacterium* isolates in this study except for R53, exhibited significant cellulase activity that could improve biomass degradation efficiency through a biological process.

In addition, amylase activity was also demonstrated in all isolates, with the highest level found in R63 (S1 Fig). Consequently, R63 designated as *Thermoanaerobacterium* sp. R63, was selected for its high extracellular hydrolytic enzymes, and subsequently used in the following studies. Although acid production was indicated in most of the isolates against Avicel (Table 2), no enzymatic activity against Avicel was detected in any of the isolates. Undetectable level of Avicelase could be owing to insufficient reaction time, since hydrolysis of insoluble substrate usually takes longer reaction time. No growth of the six isolates was observed when filter paper was supplied as a sole carbon source, implying that none of the isolates containing FPCase activity.

### Optimal growth condition of *Thermoanaerobacterium* sp. R63

**Fig 3:**
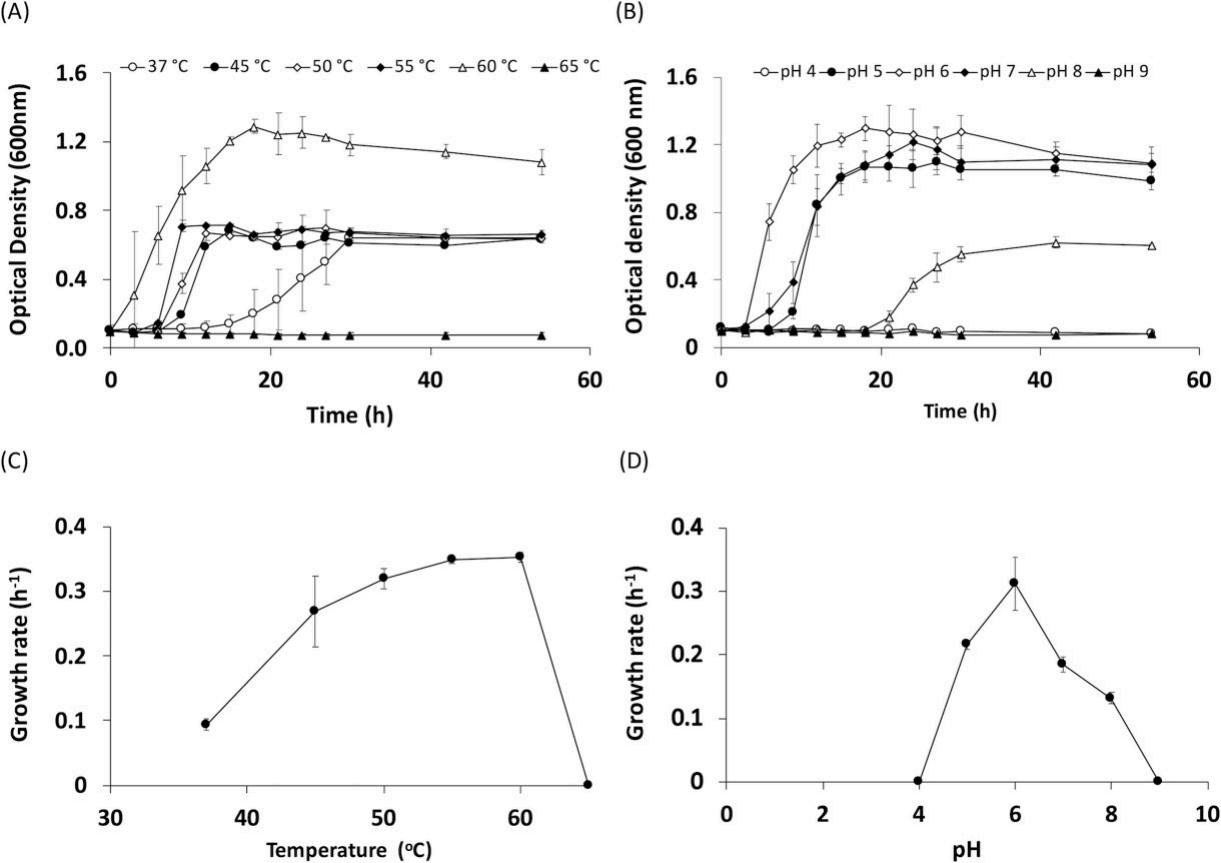
Growth characteristic of *Thermoanaerobacterium* sp. R63. *Thermoanaerobacterium* sp. R63 was grown in T6 medium supplemented with 1% glucose. Growth of the bacteria was followed up to 54 h, with an interval of 2 h during the first 20 h under different temperature (A), and pH (B). Growth rate of the bacteria was calculated from slope of natural log of OD_600_ at the linearity versus time under different temperature (C), and pH (D). Data were evaluated from three and four independent experiments for temperature and pH, respectively. Error bars in graphs represent standard deviation of the data.

The optimum growth conditions of *Thermoanaerobacterium* sp. R63 were determined at various temperatures and pH. The highest cell density was observed to reach 1.2–1.3 (OD_600_) when incubating at 60°C, which was twice as much as those cultured at other temperatures. At 37°C, the bacteria exhibited an extended lag phase, entering its exponential phase at 15 h (Fig 3A). The optimal growth temperature of *Thermoanaerobacterium* sp. R63 was determined to be 60°C (Fig 3C). No growth of the bacterium was observed after the temperature was increased to 65°C. For the effects of pH shown in Fig 3B and 3D, the optimum pH for growth was at 6.0, where the bacteria could survive at pH range from 5.0 to 8.0. The highest cell density was achieved when the medium pH was adjusted to 6.0. The bacterial growth was not observed at both pH 4.0 and 9.0. *Thermoanaerobacterium* sp. R63 shared similarity on growth characteristic with several previous reports from genus *Thermoanaerobacterium*, such as *T*. *thermosaccharolyticum*, *T*. *aotearoense* and *T*. *saccharolyticum* [42,43], but cellulolytic activity had never been reported from these bacteria. Considering that a system for genetic manipulation is currently established, and natural competence was discovery in *Thermoanaerobacterium* [44,45], genetic engineering of *Thermoanaerobacterium* sp. R63 could further enhance the ability of lignocellulosic biomass degradation of the bacteria to achieve all-in-one biomass-to-energy conversion under extreme temperature conditions.

### Effects of temperature and pH on cellulase and xylanase activities

**Fig 4:**
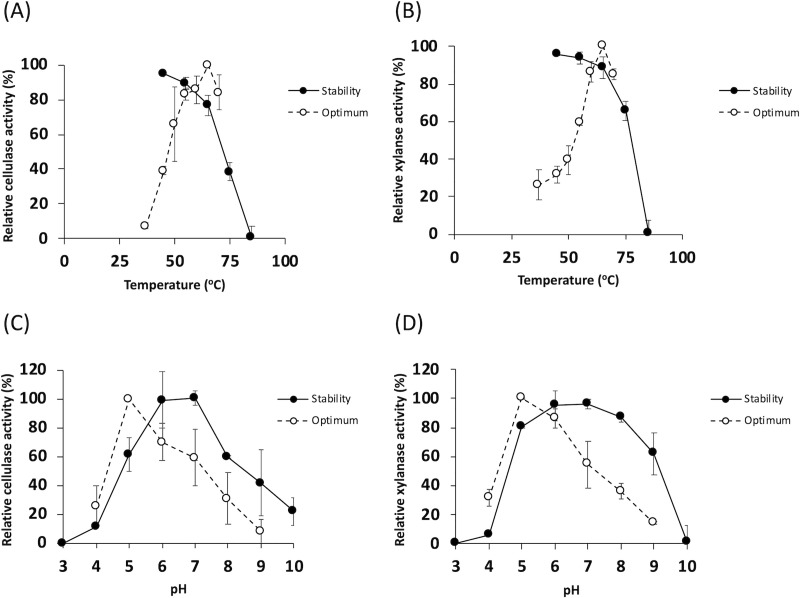
Effect of pH and temperature on crude enzyme. Effects of temperature (A, B) and pH (C, D) on the activity and stability of the crude enzymes from *Thermoanaerobacterium s*p. R63 were evaluated. Enzymatic activities were investigated by measuring reducing sugar product using DNS method. Relative activity represents specific activity in a given condition compared to the optimum condition. Data were evaluated from three independent experiments. Error bars in graphs represent standard deviation of data.

Cellulase and xylanase activities of *Thermoanaerobacterium* sp. R63 were determined under different temperatures as shown in Fig 4A and 4B. At 37°C, limited cellulase and xylanase activities were detected. The activities gradually increased when temperature was increased from 45 to 60°C, and the highest activities were achieved at 65°C. At 70°C, interestingly, more than 80% of the cellulase and xylanase activities of *Thermoanaerobacterium* sp. R63 were still detectable. At 75°C, more than 50% of xylanase activities were even detected, while approximately only 40% of the cellulase activities were detected. Thermal stability of cellulase and xylanase of the isolate was investigated by prior heating crude enzymes at different temperatures between 45 and 85°C for 60 min before performing activity assay. Stability of cellulase and xylanase of the bacteria gradually declined during the incubation at 45, 55, and 65°C. Hydrolytic activity in *Thermoanaerobacterium* sp. R63 was completely diminished when the crude enzymes were incubated at 85°C for 60 min. Effect of temperature on hydrolytic activity of *Thermoanaerobacterium* sp. R63 was consistent with other studies of thermophilic bacteria; *C*. *thermocellum*, *Bacillus licheniformis*, and *T*. *calidifontis* RX-1 [32,46,47]. With their intrinsic stability and activity at high temperatures, thermophilic enzymes could be inferred to a possibility of prolonged storage and low activity loss during long-time processing under an elevated temperature condition. Cellulase and xylanase of *Thermoanaerobacterium* sp. R63 showed the optimal pH at 5.0 (Fig 4C and 4D). At pH 9.0, a constant decrease of cellulase and xylanase activities was observed and exhibited pertaining activities below 20%. Effect of pH on hydrolytic activities of *Thermoanaerobacterium* sp. R63 seemed to be in parallel with thermostable xylanase produced by other *Thermoanaerobacterium*, and likely with thermostable cellulase from *Geobacillus* and *Bacillus* [48].

### Hydrolytic products of the crude enzyme against CMC and xylan

Catalytic products resulted from hydrolysis of the crude enzyme of *Thermoanaerobacterium* sp. R63 toward CMC and xylan were analyzed using HPLC. Glucose and cellobiose concentrations were detected from CMC hydrolysis; whereas, for xylan degradation, xylose sugar was observed as a main hydrolytic product (S2 Fig). Hydrolytic enzymes of *Thermoanaerobacterium* sp. R63, therefore, were capable of producing mono- and disaccharide from the complex polysaccharides in relation to the biomass substrate.

### Metagenomic analysis of bacterial community

**Fig 5:**
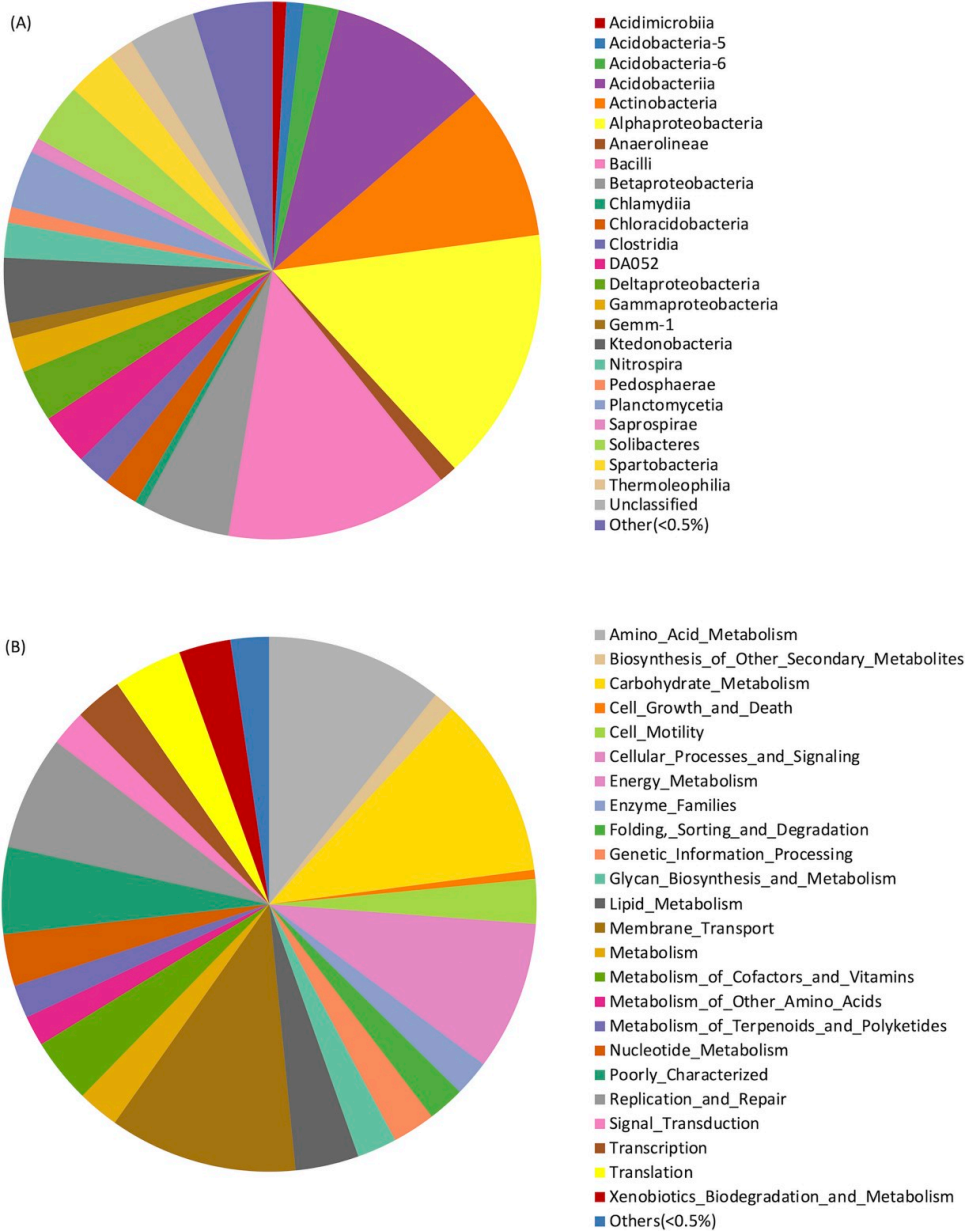
Bacterial community composition and predicted function. (A) Class-level relative abundance of bacteria in the soil sample. (B) KOs-prediction of function analysis based on 16S rRNA using PICRUSt.

Metagenomic analysis were performed to address complexity of bacterial community in the sampled tropical dry deciduous forest soil. The analysis revealed that majority of the bacteria found in our soil samples belong to the class of *Alphaproteobacteria*, *Bacilli*, *Acidobacteriia*, and *Actinobacteria* (Fig 5A). It was in good agreement with previous metagenomic data of bacterial communities in tropical forest soil from other sites, in both Asia and North America. It showed that the groups of bacteria predominant in tropical soil ecosystem were mostly in phylum Firmicutes, Bacteroides, Proteobacteria, and Actinobacteria [49–52]. The result also revealed that approximately 2% of the bacterial abundance was occupied by *Clostridia*. Notably, all 6 strains of bacteria isolated in this study belong to genus *Thermoanaerobacterium* and thus consolidate the existence of those bacteria in the tropical forest soil. It is logical to believe that this could be due to the specific selection pressure (potentially, high temperature) applied in the isolation process. The KO-prediction using PICRUSt showed the presence of carbohydrate metabolism at approximately of 11% implying that many bacteria could potentially exert their cellulolytic activity in the environment (Fig 5B). The prediction is very promising as there might be many other cellulolytic microbes to be found in tropical forest soil. Notably, the presence of xylanolytic activity could be speculated from the abundance of proteins in the groups of metabolism and others. Our results thus further substantiate the role of bacterial community in litter decomposition as well as circulation of nutrients in the ecosystem of tropical dry deciduous soil.

## Conclusion

*Thermoanaerobacterium* sp. R63 isolated from soil samples of tropical dry deciduous forest was capable of natural biomass degradation, particularly cassava pulp. Crude extract of the bacterium exhibited xylanase and cellulase activities at 6.17 U/mg and 1.15 U/mg, respectively. The optimal conditions for hydrolytic activity were at 65°C and pH 5.0. Hydrolytic activity was found to be relatively stable even at 75°C, and pH of 5.0–9.0. Thus, *Thermoanaerobacterium* sp. capable of carrying on fermentation reaction at high temperature and producing thermostable lignocellulosic enzymes from this study might be potentially useful candidates for efficient lignocellulosic biomass conversion.

## Supporting information

S1 Fig Amylase activity of the bacterial isolates. Crude enzymes from six bacterial isolates were prepared from bacterial cultures grown for 24 h in T6 medium supplemented with starch. Specific activity was investigated at 1h after the reaction was initiated by determining of reducing sugar produced using DNS method. Data were evaluated from four independent experiments. Error bars in graphs represent standard deviation. Statistical analysis was determined by one-way ANOVA with post-hoc Kruskal-Wallis multiple comparison test. (*) marked significant difference with p < 0.05, (**) marked significant difference with p < 0.01.
(TIF)

S2 Fig HPLC chromatogram of the product of polysaccharide degradation. Standard mixture was prepared by mixing 1 mg/ml of xylose, glucose, and cellobiose (A). Control; crude enzyme of *Thermoanaerobacteirum* sp. R63 cultured in CMC (B) and xylan (C). Hydrolytic products of the crude enzyme incubated with CMC (D) and xylan (E).
(TIFF)

S1 File Statistical analysis of enzymatic activity of the bacterial isolates. Statistical analysis of cellulase and xylanase activities of six isolates was determined by two-way ANOVA with post-hoc Tukey’s multiple comparison test using GraphPad Prism 8.4.2.
(CSV)
