# Supplementary figures and images for: Isolation and characterization of thermophilic cellulose and hemicellulose degrading bacterium, Thermoanaerobacterium sp. R63 from tropical dry deciduous forest soil
Source: PLoS One. 2020 Jul 23;15(7):e0236518. doi: 10.1371/journal.pone.0236518 (PMC7377481; doi:10.1371/journal.pone.0236518)

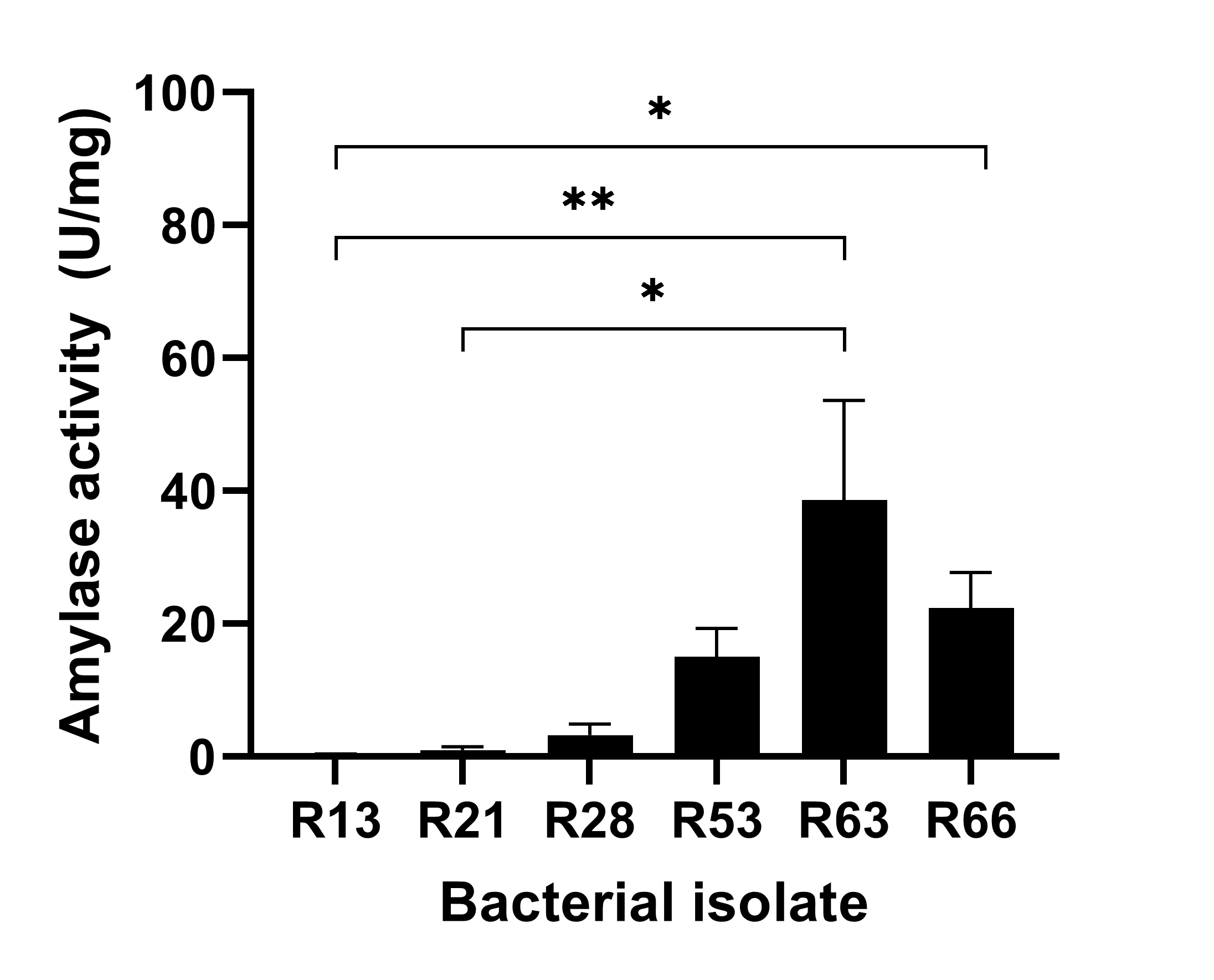

Supplement: S1 Fig — Crude enzymes from six bacterial isolates were prepared from bacterial cultures grown for 24 h in T6 medium supplemented with starch. Specific activity was investigated at 1h after the reaction was initiated by determining of reducing sugar produced using DNS method. Data were evaluated from four independent experiments. Error bars in graphs represent standard deviation. Statistical analysis was determined by one-way ANOVA with post-hoc Kruskal-Wallis multiple comparison test. (*) marked significant difference with p < 0.05, (**) marked significant difference with p < 0.01. (TIF) [file pone.0236518.s001.tif]

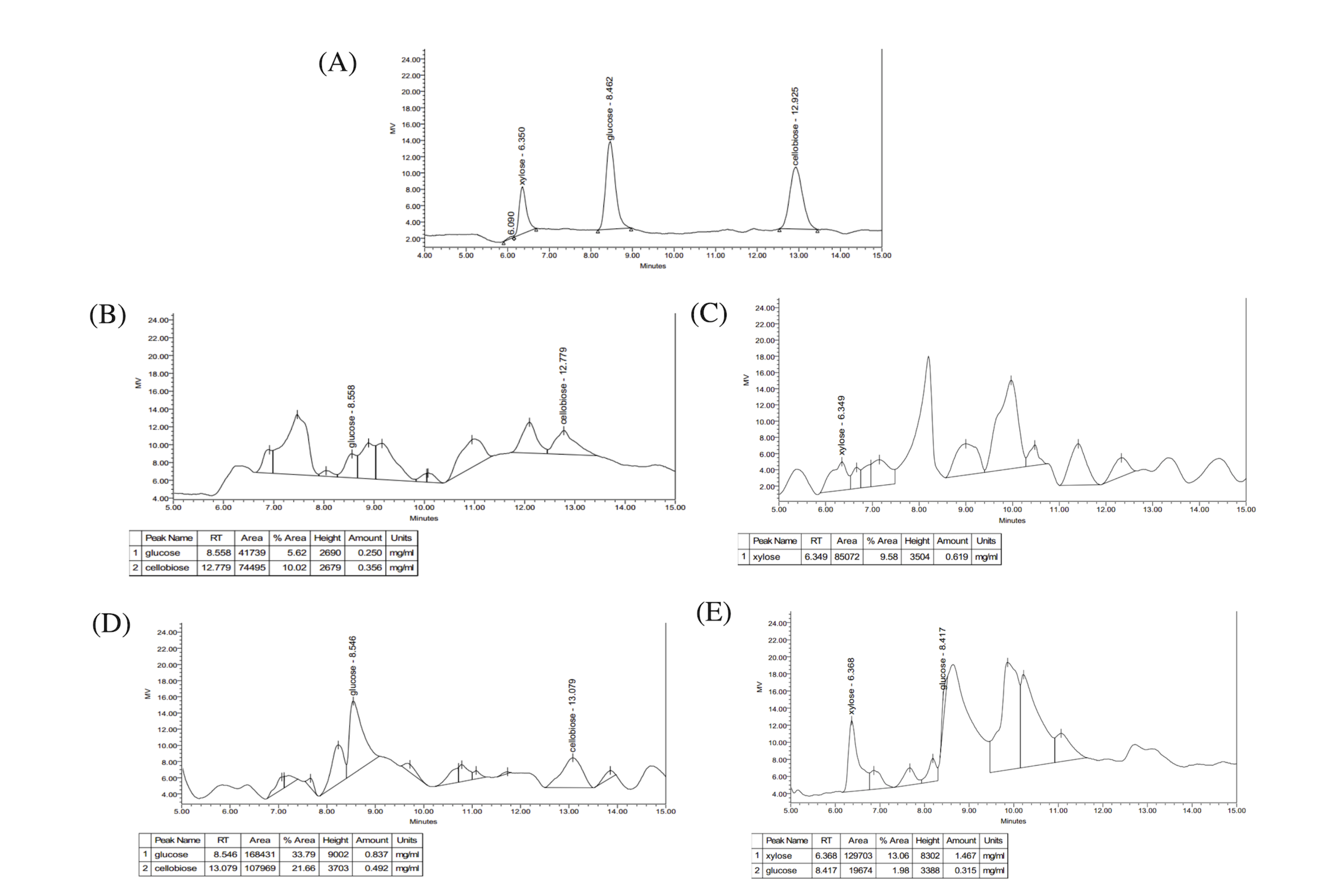

Supplement: S2 Fig — Standard mixture was prepared by mixing 1 mg/ml of xylose, glucose, and cellobiose (A). Control; crude enzyme of Thermoanaerobacteirum sp. R63 cultured in CMC (B) and xylan (C). Hydrolytic products of the crude enzyme incubated with CMC (D) and xylan (E). (TIFF) [file pone.0236518.s002.tiff]
